# Internet-based transdiagnostic treatment for emotional disorders in Arabic- and Farsi-speaking refugees: study protocol of a randomized controlled trial

**DOI:** 10.1186/s13063-023-07845-5

**Published:** 2024-01-02

**Authors:** Johanna Boettcher, Manuel Heinrich, Maria Boettche, Sebastian Burchert, Heide Glaesmer, Euphrosyne Gouzoulis-Mayfrank, Carina Heeke, Martina Hernek, Christine Knaevelsrud, Alexander Konnopka, Louisa Muntendorf, Hannah Nilles, Laura Nohr, Steffi Pohl, Sophia Paskuy, Isabelle Reinhardt, Susan Sierau, Nadine Stammel, Christina Wirz, Babette Renneberg, Birgit Wagner

**Affiliations:** 1 Clinical Psychology and Psychotherapy, Freie Universitaet Berlin, Berlin, Germany; 2 Clinical Psychology and Psychotherapy, Psychologische Hochschule Berlin, Berlin, Germany; 3 Clinical Psychological Intervention, Freie Universitaet Berlin, Berlin, Germany; 4 Medical Psychology and Medical Sociology, University of Leipzig, Leipzig, Germany; 5 Section of Healthcare Research, LVR-Institute for Research and Education, Cologne, Germany; 6 Clinical Psychology and Psychotherapie, Freie Universitaet Berlin, Berlin, Germany; 7 Health Economics and Health Services Research, University Medical Center Hamburg-Eppendorf, Hamburg, Germany; 8 Clinical Psychology and Psychotherapy, Medical School Berlin, Berlin, Germany; 9 Methods and Evaluation/Quality Assurance, Freie Universitaet Berlin, Berlin, Germany

**Keywords:** Cognitive-behavioral, Common elements treatment approach, Refugee, App based, Tailored

## Abstract

**Background:**

Refugee populations have an increased risk for mental disorders, such as depression, anxiety, and posttraumatic stress disorders. Comorbidity is common. At the same time, refugees face multiple barriers to accessing mental health treatment. Only a minority of them receive adequate help. The planned trial evaluates a low-threshold, transdiagnostic Internet-based treatment. The trial aims at establishing its efficacy and cost-effectiveness compared with no treatment.

**Methods:**

*N* = 131 treatment-seeking Arabic- or Farsi-speaking patients, meeting diagnostic criteria for a depressive, anxiety, and/or posttraumatic stress disorder will be randomized to either the intervention or the waitlist control group. The intervention group receives an Internet-based treatment with weekly written guidance provided by Arabic- or Farsi-speaking professionals. The treatment is based on the Common Elements Treatment Approach (CETA), is tailored to the individual patient, and takes 6–16 weeks. The control group will wait for 3 months and then receive the Internet-based treatment.

**Discussion:**

The planned trial will result in an estimate of the efficacy of a low-threshold and scalable treatment option for the most common mental disorders in refugees.

**Trial registration:**

German Registry for Clinical Trials DRKS00024154. Registered on February 1, 2021.

**Supplementary Information:**

The online version contains supplementary material available at 10.1186/s13063-023-07845-5.

## Administrative information

**Table:** 

Title {1}	Internet-based transdiagnostic treatment for emotional disorders in Arabic- and Farsi-speaking refugees: study protocol of a randomized controlled trial
Trial registration {2a and 2b}	The trial is registered at the German Clinical Trials Registry GCTR: DRKS00024154. The trial was registered on 2021–02-01 and adapted on 2023–11-08
Protocol version {3}	2023–10-23, version 2
Funding {4}	This study is funded by the German Federal Ministry of Education and Research (01EF1806F)
Author details {5a}	Johanna Boettcher, Freie Universitaet Berlin, Clinical Psychology and Psychotherapy & Psychologische Hochschule Berlin, Clinical Psychology and Psychotherapy (corresponding author), johanna.boettcher@fu-berlin.de Manuel Heinrich, Freie Universitaet Berlin, Division of Clinical Psychological Intervention, manuel.heinrich@fu-berlin.de Maria Boettche, Freie Universitaet Berlin, Division of Clinical Psychological Intervention, maria.boettche@fu-berlin.de Sebastian Burchert, Freie Universitaet Berlin, Division of Clinical Psychological Intervention, s.burchert@fu-berlin.de Heide Glaesmer, Medical Psychology and Medical Sociology, University of Leipzig, heide.glaesmer@medizin.uni-leipzig.de Euphrosyne Gouzoulis-Mayfrank, Section of Healthcare Research, LVR-Institute for Research and Education, Euphrosyne.Gouzoulis-Mayfrank@lvr.de Carina Heeke, Freie Universitaet Berlin, Division of Clinical Psychological Intervention, carina.heeke@fu-berlin.de Martina Hernek, Freie Universität Berlin, Division of Clinical Psychology and Psychotherapie, martina.hernek@fu-berlin.de Christine Knaevelsrud, Freie Universitaet Berlin, Division of Clinical Psychological Intervention, christine.knaevelsrud@fu-berlin.de Alexander Konnopka, University Medical Center Hamburg-Eppendorf, Health Economics and Health Services Research, a.konnopka@uke.de Louisa-Kristin Muntendorf, University Medical Center Hamburg-Eppendorf, Health Economics and Health Services Research, l.muntendorf@uke.de Hannah Nilles, Medical School Berlin, Clinical Psychology and Psychotherapy, hannah.nilles@medicalschool-berlin.de Laura Nohr, Freie Universitaet Berlin, Division of Clinical Psychological Intervention, laura.nohr@fu-berlin.de Steffi Pohl, Freie Universitaet Berlin, Methods and Evaluation/Quality Assurance, steffi.pohl@fu-berlin.de Sophia Paskuy, Medical School Berlin, Clinical Psychology and Psychotherapy, sophia.paskuy@medicalschool-berlin.de Isabelle Reinhardt, Section of Healthcare Research, LVR-Institute for Research and Education, Isabelle.Reinhardt@lvr.de Susan Sierau, Medical Psychology and Medical Sociology, University of Leipzig, Susan.Sierau@medizin.uni-leipzig.de Nadine Stammel, Freie Universitaet Berlin, Division of Clinical Psychological Intervention, nadine.stammel@fu-berlin.de Christina Wirz, Freie Universitaet Berlin, Clinical Psychology and Psychotherapy, c.wirz@fu-berlin.de Babette Renneberg, Freie Universitaet Berlin, Clinical Psychology and Psychotherapy, b.renneberg@fu-berlin.de Birgit Wagner, Medical School Berlin, Clinical Psychology and Psychotherapy, birgit.wagner@medicalschool-berlin.de (trial sponsor)
Name and contact information for the trial sponsor {5b}	Birgit Wagner, Medical School Berlin, Clinical Psychology and Psychotherapy, birgit.wagner@medicalschool-berlin.de
Role of sponsor {5c}	The sponsor co-developed the study design. She will supervise data collection, management, analysis and interpretation, writing of the report, and decision to submit the report. She has ultimate authority over these processes

## Introduction

### Background and rationale {6a}

Mental disorders, such as posttraumatic stress disorder (PTSD), anxiety, depressive, and substance abuse disorders, are common within refugee and migrant populations [1–3]. (Forced) migration, exposure to traumatic events, and post-migration factors all increase the vulnerability to developing mental disorders [1, 4, 5]. At the same time, treatment resources for the specific population of refugees and migrants are very limited in many host countries [6, 7]. Since 2015, approximately 2 million people sought asylum in Germany. Arabic- and Farsi-speaking individuals constitute the largest group among Asylum seekers in Germany in 2022 [8]. The number of psychotherapists in Germany speaking these languages is negligible. In addition, complicated legal regulations make it hard for therapists to get funding for the necessary interpreters. Hence, in Germany, only about 5% of the refugees with mental health problems receive adequate treatment [9]. This is significantly lower than rates in the general population [10]. Language barriers, limited knowledge of treatment options, and perceived stigma further contribute to the large gap between treatment needs and treatment uptake.

Untreated, mental disorders cause major impairments in social and occupational functioning and lead to a loss in quality of life [11]. In refugees and migrants, mental health problems may hinder the process of successfully adapting to the conditions in the host country [12]. Impairment aggravates if more than one mental disorder is present, which is rather the rule than the exception for mental disorders (e.g., [13]). Comorbidity patterns in refugee populations are less well-known than in the general population, but studies suggest that there is substantial comorbidity between PTSD and depression [4] as well as between substance abuse and other mental disorders [14].

It, therefore, seems adamant to invest in low-threshold, accessible interventions that can reach the vulnerable group of refugees and that take into account the comorbid nature of mental disorders.

Internet-based guided self-help interventions can overcome some of the obstacles associated with treatment uptake. They provide a treatment environment that is easy to access, that is less associated with the stigma of mental health treatment, and that is independent of time and location. Numerous studies show that Internet-based guided self-help interventions are effective in the treatment of PTSD, anxiety, and depression [15–17] and demonstrate non-inferiority to face-to-face treatments [18]. Mainly, Internet-based treatments have been investigated in a disorder-specific format, but there are several trials supporting the efficacy of more transdiagnostic approaches [19].

Internet-based treatments also have the potential to overcome language barriers. Treatment contents can be translated into several different languages, and online therapists and clients do not necessarily have to reside in the same area. However, Internet-based interventions for “non-Western” populations are still scarce. In two pilot studies, Kayrouz and colleagues [20, 21] tested the efficacy of an online treatment for depression and anxiety for Arabic-speaking migrants in Australia. The authors reported promising effects for a guided version of the program. In a feasibility study, Lindegaard also reported good effects of a guided program for depression and anxiety for Arabic-speaking patients in Sweden [22]. A small pilot study of a comparable program for *N* = 15 Dari- or Farsi-speaking youths showed high drop-out rates [23]. In two large-scale studies, Knaevelsrud and colleagues [24, 25] could show that a guided online program led to large changes in PTSD symptoms in patients in war and conflict zones. Although very encouraging, the focus of these trials was on PTSD, excluding other very prevalent problems such as anxiety, depression, and substance abuse. A recent study in Germany evaluated an unguided app as part of a stepped-care approach in Arabic-speaking refugees with depression [26]. The stepped-care approach proved slightly superior compared to treatment as usual. The efficacy of the app, however, was not reported separately.

The majority of refugees access the Internet via smartphones on a daily basis [27]. Developing Internet-based interventions that address the most common mental disorders and that operate on limited therapist time, while, at the same time, providing the necessary clinical support, presents a valuable alternative in situations in which support would otherwise not be available. The aim of the planned trial is therefore to test whether a transdiagnostic, Internet-based treatment is effective in treating emotional disorders in Arabic- and Farsi-speaking refugees.

For the planned trial, we will apply the Common Elements Treatment Approach [28]. CETA is a transdiagnostic, tailored intervention addressing symptoms of depressive and anxiety disorders, PTSD, and substance abuse. CETA was evaluated as a face-to-face treatment in three large RCTs in samples of Burmese refugees [29], survivors of torture and militant attacks in Iraq [30], and couples experiencing alcohol misuse and partner violence in Zambia [31]. CETA demonstrated large effects on post-traumatic stress symptoms, depression, anxiety, substance use, functional impairment, and aggression [29–32]. We adapted treatment contents to the target population of refugees residing in Germany. We followed the conceptual framework and reporting guidelines for cultural adaptation of interventions for common mental disorders [33]. The adaptation process, including expert interviews and focus groups, showed that the CETA manual seemed culture sensitive to the target population, and only surface adaptations were made [34].

### Objectives {7}

In the planned trial, we will test whether a guided, Internet-based version of CETA (CETA-I) is effective compared to no treatment, using a waitlist control condition (WL). We hypothesize that the Internet-based treatment will decrease mental distress, as well as symptoms of anxiety, depression, and post-traumatic stress. We will also investigate potential predictors and mediators. As therapy modules will be differently assigned to patients depending on their primary symptoms, we will specifically focus on baseline symptoms as moderators of the treatment effect. We will additionally explore potential mediators for the relation of treatment and outcome, such as working alliance or CBT skills acquisition. We will also examine cost-effectiveness.

### Trial design {8}

The planned trial applies a randomized controlled design, testing the superiority of an intervention condition (CETA-I) compared to waitlist control.

## Methods: participants, interventions, and outcomes

### Study setting {9}

The randomized controlled trial (RCT) will be realized within an alliance of related subprojects. One of the subprojects focused on adapting CETA to the target population (see item 6a), one focused on translating and evaluating measures of psychopathology for the population, and one evaluated an unguided version of the transdiagnostic online treatment in an inpatient setting.

The trial will take place in Germany.

### Eligibility criteria {10}

We will apply a two-step screening procedure. First, participants will be invited to complete online questionnaires, including the Hopkins Symptom Checklist-25 (HSCL, [35] and the PTSD Checklist for DSM-5 (PCL-5, [36]). Participants scoring above the cutoff on the anxiety and/or depression subscale of the HSCL (> 1.75, [37]) and/or above the cutoff on the PCL-5 (> = [38]) will be invited to take part in a telephone-administered clinical interview. Trained and supervised Arabic- and Farsi-speaking interviewers will administer the Mini-DIPS, a brief, adapted, open-access version of the Anxiety and Related Disorders Interview Schedule (ADIS) based on DSM-5 [39] plus the more elaborate PTSD section of the DIPS [40]. As there are no Arabic or Farsi versions available, we will use our own translations of the Mini-DIPS, realized according to the WHO standards [41].

Inclusion criteria are as follows: (a) age ≥ 18 years; (b) Internet access; (c) primary diagnosis of a depressive disorder (major depressive disorder, persistent depressive disorder), anxiety disorder (generalized anxiety disorder, panic disorder, agoraphobia, social anxiety disorder), or PTSD according to DSM-5; (d) no current psychotic or bipolar disorder; (e) no severe substance use disorder (AUDIT score ≤ 15 for men, ≤ 13 for women; DUDIT score ≤ 25); (f) no serious suicidal ideation at baseline and no suicide attempt within the last 12 months; and (g) no ongoing psychotherapy or unstable psychotropic medication 3 months prior to the study.

### Who will take informed consent? {26a}

A study website will inform about the intervention and the goal and design of the study. Potential participants will be invited to download the study app. Within the app, participants will receive detailed information (see item 32) and will be asked to provide informed consent online.

### Additional consent provisions for collection and use of participant data and biological specimens {26b}

Within the registration process, participants will be asked whether they agree to be contacted for another ancillary study concerning dropout in interventions for refugees. If they agree and drop out of treatment, they will be contacted and will then receive detailed information and be asked to provide informed consent via postal mail.

### Interventions

#### Explanation for the choice of comparators {6b}

A transdiagnostic, cognitive-behavioral Internet-based intervention (CETA-I) will be compared to a waitlist control condition. Comparisons to waitlists are ethical and necessary to establish the efficacy of a new intervention.

#### Intervention description {11a}

The treatment is based on a version of CETA [28] that has been adapted to the context of Arabic- and Farsi-speaking refugees in Germany [34]. CETA includes several modules that are tailored to the patient’s needs. Table 1 gives an overview of the CETA modules applied in the current trial (for a detailed description, please see [28]). Modules differ regarding the number of sessions they encompass.

**Table 1:** CETA modules and dosing limits

***Module***	*Target*	*Sessions*
**Psychoeducation and engagement**	Treatment motivation and barriers	1–1.5
**Cognitive restructuring 1**	Dysfunctional cognitive patterns	1–1.5
**Cognitive restructuring 2**	Dysfunctional cognitive patterns	1.5–3.5
**Imaginal and in vivo exposure**	Avoidance/intense fears of specific situations/external and internal cues	2–4
**Behavioral activation**	Low drive, social withdrawal, anhedonia	2–4
**Trauma exposure**	Posttraumatic symptoms	3–5
**CBT for substance abuse**	Problematic alcohol/drug use or gambling	2–4
**Problem-solving**	Addressing real-life solvable problems and problems with treatment engagement	1–3
**Safety planning**	Suicidal ideation, aggression, & violence	1–3
**Finishing steps**	Relapse prevention	0.5–1

## Adaptations to the original CETA manual

For the current trial, we made the following adaptations to the CETA manual. In the two cognitive restructuring modules, we excluded two skills (providing facts and logical questioning). We also decided to start every first cognitive restructuring two sessions with the friend/family role-play skill. For the exposure module, we defined three different types of exposure (imaginative exposure, in vivo exposure, and interoceptive exposure) and will tailor these to the patients. Furthermore, we opened the substance abuse module for patients reporting gambling problems. In the problem-solving module, we specifically probe for problems related to postmigration stressors like discrimination.

## Tailoring

All participants receive the psychoeducation and engagement module, the cognitive restructuring modules, and the finishing steps module. In addition, as follows:

- Participants scoring above the cutoff on the PCL-5 (> = 33) and/or fulfilling diagnostic criteria for PTSD qualify for the trauma exposure module.
- Participants scoring above the cutoff of the depression subscale of the HSCL-25 (> 1.75) and/or fulfilling diagnostic criteria for a depressive disorder qualify for the behavioral activation module.
- Participants scoring above the cutoff of the anxiety subscale of the HSCL-25 (> 1.75) and/or fulfilling diagnostic criteria for an anxiety disorder qualify for the exposure module.
- Participants indicating mild to moderate addiction problems by scoring x ≤ 13 or more for women and x ≤ 15 or more for men on the AUDIT and 3 ≤ x ≤ 25 for women and 6 ≤ x ≤ 25 for men on the DUDIT, or indicating addictive behaviors on screening for behavioral addictions, and/or fulfilling diagnostic criteria for a mild to moderate substance-related or addictive disorder qualify for the substance abuse module.
- Participants endorsing a single question asking whether they are facing a major problem in their daily life that has not yet been addressed in therapy and participants who indicate that there is some problem compromising their adherence to therapy qualify for the problem-solving module.
- Participants who (a) indicate (mild) suicidal ideation on the HSCL-25 during the diagnostic interview, and/or (b) participants who report being a potential threat to others, and/or (c) participants who report being exposed to domestic violence (both (b) and (c) assessed by a single question, rated on a 4-point Likert scale), qualify for the safety module.

In a clinical case conference, a sequence of modules is defined for every patient before treatment, integrating information from the diagnostic interview, baseline questionnaire data, and the patient’s description of his/her main problem. Standard flows for specific (combinations of) diagnoses are referred to (see Appendix 1). A maximum of two types of disorder-specific modules will be administered. Depending on the individual symptom profile, participants receive 5–10 modules.

During treatment, dosage and sequence of modules can also be adapted to the patient’s progress. However, we only allow for specific adaptions. Only modules on safety planning, problem-solving, and substance use can be added when related issues arise. Furthermore, a maximum number of sessions per module is pre-defined (see Appendix 1). Participants will receive a minimum total of 6 sessions and a maximum total of 16 sessions.

Participants can access the online program as an app on their smartphone/tablet and/or as a browser-based version on their desktop computer. Participants are asked to complete one session per week. In each session, participants receive psychoeducation and complete exercises. They receive short written feedback from an online therapist within 48 h after completing a session. Online therapists are trained and supervised Arabic- or Farsi-speaking professionals (bachelor’s or master’s degree in psychology or related subjects). A 2-day training workshop will include training in (a) administering the CETA components; (b) handling the online platform; (c) providing brief, constructive feedback; and (d) enabling crisis management. Supervision will be provided on a biweekly basis.

### Supervision

Supervisors and trainers will be licensed CBT therapists or M.Sc. psychologists in advanced CBT training. Training and supervision in this trial deviate from the standard amount of training in previous CETA trials. First, the amount of training for therapists is lower than in previous trials (2 days instead of 10 days). Second, the supervisors and trainers in our trial are not certified CETA trainers/supervisors. While our trainers and supervisors have received extensive training in providing CETA training and supervision by the authors of CETA (Laura Murray & Kristie Metz), they did not complete all the requirements to qualify as certified CETA trainers/supervisors due to time restraints within the study.

#### Criteria for discontinuing or modifying allocated interventions {11b}

A participant may withdraw from the study at any time, for any reason, specified or unspecified, and without penalty or loss of benefits. Furthermore, the principal investigator has the right to discontinue the treatment of participants in case of the following: (a) adverse events (e.g., suicide attempt, severe major depression) that impede treatment or impair the interpretability of the study results or (b) reasons precluding attendance of scheduled treatment modules. Date of discontinuation, all recorded results at that time, and, if known, the reasons for discontinuation will be documented. All participants will be followed up at each assessment point after discontinuation according to the intention-to-treat principle.

We will not allow for the modification of allocated interventions.

#### Strategies to improve adherence to interventions {11c}

## Therapists

Online therapists will receive biweekly supervision. Adherence will further be supported by pre-written feedback messages that online therapists adapt to the individual patient. Adherence will be assessed by analyzing a random selection of written feedback messages. Messages will be analyzed in a qualitative content approach, based on previous work on therapist behavior in guided Internet-based interventions (e.g., [42]). The amount of non-adherent therapist behavior will be assessed.

## Participants

Participants who indicate that there is some problem compromising their adherence to therapy will receive the problem-solving module and work with their therapist to overcome these obstacles.

### Relevant concomitant care permitted or prohibited during the trial {11d}

Participants can continue their respective pharmacological treatment but are requested to not change the dosage or take up additional psychological treatment throughout the treatment/waitlist period.

#### Provisions for posttrial care {30}

Participants in the waitlist control condition receive access to the Internet-based treatment after a waiting period of 3 months.

#### Outcomes {12}

Patient outcome measures consist of self-report questionnaires, which are assessed at baseline (week 0), at week 1, at mid-treatment (week 5), at post-treatment (last session/week 12), and, for the active condition, at 3-month (week 24) and 6-month follow-up (week 36). The primary endpoint is at post-treatment (12 weeks after randomization for the waitlist group).

All questionnaires will be presented via the platform that also hosts the CETA-I intervention. See Table 2 and the section “Data collection and management” for details of measurements at all time points.

**Table 2:** Schedule of enrolment, interventions, and assessments

		**Enrolment**	**Allocation**	**Post-allocation**				
**Assessment/activity**		**Baseline**		**Week 1**	**Week 5**	**Post**	**3-month follow-up**	**6-month follow-up**
**Enrolment**	Eligibility screen	x						
	Informed consent	x						
	Allocation		x					
**Intervention**	CETA-I							
	Waitlist							
**Questionnaires for patients**	Sociodemographics	X						
	Healthcare utilization (FIMA & FIMPsy)	X				X		X
	Mental distress, anxiety, depression (HSCL-25)	X			X	X	X	X
	Posttraumatic stress symptoms (PCL-5)	X			X	X	X	X
	Alcohol use (AUDIT)	X			X	X	X	X
	Drug use (DUDIT)	X			X	X	X	X
	Behavioral addictions (own instrument)	X			X	X	X	X
	Health-related quality of life (EQ-5D-5L)	X				X	X	X
	Social support (OSSS-3)	X				X	X	X
	Self-efficacy (GSE)	X				X	X	X
	CBT skills (CBT-SQ)	X				X		
	Outcome expectations (three items)			X				
	Postmigration living difficulties (PMLD-short version)			X				
	Working alliance (WAI-I)				X	X		
	Treatment satisfaction (CSQ-I)					X		
	Negative Effects Questionnaire (NEQ)					X		
	**Weekly assessments**							
	Mental distress (PHQ-4)							
	PTSD (PCL-5-short version)							
	Drug and alcohol use							
	Suicidal ideation, domestic violence, aggressive behaviors (single items)							

#### Participant timeline {13}

**Fig. 1:**
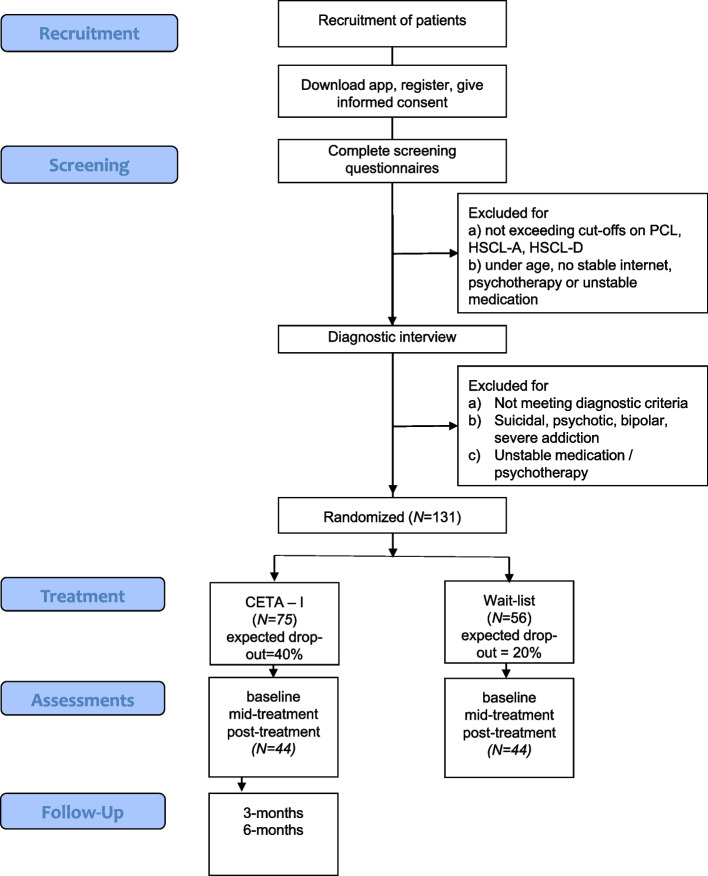
Anticipated participant flow

Figure 1 depicts the participants’ anticipated flow.

#### Sample size {14}

A pragmatic viewpoint was taken in planning the sample size. We assumed that the difference between active treatment and waitlist conditions is between *d* = 0.6–0.8 [30]. Both effects flag clinical meaningful between-group differences. Using an alpha of 5% (two-sided test), randomizing *N* = 44 individuals to each study arm would result in a power of 80% (> 95%) for comparing the WL against the CETA-I condition with *d* = 0.6 (*d* = 0.8). To avoid that the sample becomes too small, we used *n* = 44 for further planning. Two RCTs on CETA [29, 30] reported moderate attrition rates in the treatment (1 to 18%) and waitlist group (4 to 23%). In the RCT on online treatments of Arabic-speaking patients [24], patients showed an attrition rate of 40%. To compensate for dropout, we conservatively assume an attrition rate of 20% in the waitlist group and 40% in the CETA-I condition. Thus, we allocate *N* = 131 individuals (CETA-I: 75; waitlist: 56).

#### Recruitment {15}

Participants will be mainly recruited through the Internet using Google and Facebook ads and through postings in relevant social media groups and mental health forums.

### Assignment of interventions: allocation

#### Sequence generation {16a}

A randomization mechanism is hard coded into the online platform that hosts the intervention.

#### Concealment mechanism {16b}

After participants complete the baseline assessment and diagnostic interview, the platform randomizes them to one of the two groups.

#### Implementation {16c}

We will apply a simple randomization procedure, with no blocks or stratification. The randomization list will be prepared by an independent investigator unrelated to the study using the sealed envelope web app. The list will be transferred directly to the software development agency which will integrate the list into the randomization tool of the platform. Study investigators, therapists, or others have no access to the randomization list or tool and cannot influence randomization at any point.

### Assignment of interventions: blinding

#### Who will be blinded? {17a}

Due to the nature of psychotherapeutic treatment trials, neither participants therapists nor study personnel can be blind to treatment allocation.

#### Procedure for unblinding if needed {17b}

The design is open label, so unblinding for study coordinators will not occur.

### Data collection and management

#### Plans for assessment and collection of outcomes {18a}

Table 2 gives an overview of the assessment plan. The *primary outcome* is mental distress, assessed by the total score of the HSCL-25, self-report version. The 25 items of the HSCL assess mental health symptoms on a 4-point Likert scale. The questionnaire has been validated in refugee populations and showed good psychometric properties [43, 44].

#### Secondary outcomes

As secondary outcomes, we will assess *depression* with the 15-item HSCL-25 *depression* subscale and *anxiety* with the 10-item HSCL-25 anxiety subscale. The use of the total score and the subscale scores is supported by results on the factorial validity of the HSCL-25 in Arabic-speaking samples. Two studies show a clear distinction between depression and anxiety factors as well as an overall factor capturing mental distress [44, 45]. While one study [44] found two “anxiety” factors (seven items loading on a general anxiety factor and three factors loading on a “phobic anxiety” factor), it seems justified to combine these two factors when investigating anxiety disorders in a transdiagnostic approach.

## PTSD

At pre-assessment, participants will indicate whether they have ever experienced a traumatic event. *Posttraumatic symptoms* will then be assessed with the PCL-5 [36]. The PCL-5 assesses PTSD symptoms according to DSM-5 with 20 items (total score range 0–80). The Arabic and Farsi versions of the PCL-5 showed good psychometric properties [46, 47].

## Addictions

Problematic alcohol and drug use will be assessed by the 10-item AUDIT [48–50] and 11-item DUDIT [51, 52] questionnaires respectively. The Arabic AUDIT and DUDIT showed good reliability and validity [50, 52] as did the Farsi versions [53, 54]. For assessing *pathological gambling* and other addictive behaviors, we developed six items, rated on a 5-point Likert scale, depicting DSM-5 criteria of pathological gambling.

To monitor *treatment progress over time*, we will include a brief measure on mental distress (Patient Health Questionnaire-4 (PHQ-4) [55]); the 4-item short version of the PCL-5 [56]; three questions screening for suicidal ideation, domestic violence, and aggressive behaviors; and four questions depicting the use of and craving for alcohol or drugs as weekly measures.

To assess *health-related quality of life*, we will administer the EQ-5D-5L. On six items, the EQ-5D-5L assesses general health and quality of life in five different domains (e.g., mobility, and daily activities). It is available in Arabic and Farsi and shows excellent psychometric properties across a broad range of populations, conditions, and settings [57].

We will assess *patient satisfaction* with an adapted version of the Client Satisfaction Questionnaire for Internet-based treatments (CSQ-I) [58]. Potential *side effects* of treatment will be assessed using the 20-item version of the Negative Effects Questionnaire [59].

### Predictors and moderators of treatment outcome

To assess potential predictors and moderators of treatment outcomes, we will use the baseline scores of the secondary outcomes measuring PTSD, depression, anxiety, and substance abuse symptoms as described above. We will also assess sociodemographic and asylum-related variables (e.g., length of stay, asylum status), post-migration stressors (using 11 items of the Post-Migration Living Difficulties Scale (PMLD)-Short Version [60, 61]), self-efficacy (using the 12-item General Self-efficacy Scale (GSE) [62]), and social support (using the 3-item Oslo Social Support Scale (OSSS-3) [63]).

#### Mediators of treatment outcome

As potential mediators, we will assess working alliance (using an adapted version for Internet-based treatments (WAI-I) [64]), outcome expectations and perceived barriers [65], and CBT skills (using the Cognitive-Behavioral Therapy Skills Questionnaire (CBT-SQ) [66].

#### Cost-effectiveness

To assess *costs*, healthcare utilization will be measured by selected questions from the questionnaires FIMA [67] and FIMPsy [68]. To calculate costs, the measured healthcare utilization will be monetarily valued using standardized unit costs for Germany [69, 70]. Intervention costs will be calculated using a bottom-up approach based on interviews with study personnel on resource use for the development, implementation, and use of the intervention. Resources will be monetarily valued using labor costs for productivity and market prices for goods.

Questionnaires that are not available in Arabic or Farsi will be translated according to the WHO criteria [41].

#### Plans to promote participant retention and complete follow-up {18b}

Participants who end or drop out from treatment will still be invited to participate in the assessments. Participants will be compensated for completing the assessments with up to 60€.

#### Data management {19}

The data safety management plan was approved by the Ethical Committee of the Medical School Berlin (MSB-2023/131). All data is gathered online and will be recorded automatically. Data will be stored on a secure server.

#### Confidentiality {27}

Participants will register on the study platform and will be invited to provide a telephone number (for the diagnostic interview as well as for safety calls, see item 22) and an email address to recover their account. All data is stored on the secure study platform. Data export (excluding personal data) is only available to investigators.

Participants will receive extensive information on what data is assessed, how it is transferred, and where and how long it is stored. Participants provide informed consent.

#### Plans for collection, laboratory evaluation, and storage of biological specimens for genetic or molecular analysis in this trial/future use {33}

N/a: No biological specimens will be collected.

### Statistical methods

#### Statistical methods for primary and secondary outcomes {20a}

Differences in means between the treatment and waitlist conditions on the primary outcome measure (HSCL-25 total score, at post-assessment) and on secondary outcome measures will be tested (superiority) using baseline-adjusted regression models. Unstandardized and standardized between-group differences (*d*) in the primary and secondary outcomes will be reported with appropriate confidence intervals. Moreover, we report dropout rates and provide descriptive statistics on treatment adherence. Moderators and mediators of treatment response will be investigated using regression models.

For the economic evaluation, a cost-utility analysis of CETA-I compared to WL from a healthcare systems perspective will be performed using a modeling approach. A Markov model will be constructed using data on effectiveness (measured by HSCL-25), costs (measured using selected variables from FIMA, FIMPsy), and health-related quality of life (measured by EQ-5D-5L) from the study RCT, complemented by data from the literature. The primary outcome of the economic evaluation will be the incremental cost-effectiveness ratio of CETA-I compared to WL. To analyze uncertainty, univariate and probabilistic sensitivity analyses will be conducted. In addition, budget-impact analyses for CETA-I will be carried out using the same model to estimate the monetary budget impacts of different scenarios. Necessary cost data will be taken from the cost-utility analysis, complemented by data from the literature.

#### Interim analyses {21b}

No interim analysis is planned in this trial.

#### Methods for additional analyses (e.g., subgroup analyses) {20b}

To evaluate differential treatment effects, the interaction effects of moderators with treatment on the outcome will be tested. Exploratory mediation analyses will be conducted.

#### Methods in analysis to handle protocol non-adherence and any statistical methods to handle missing data {20c}

For the ITT analyses, missing data will be dealt with using multiple imputations, which is considered a gold standard [71]. Imputation will be done under the missing at-random assumption including different predictors and moderators (as outlined in item 18a) of attrition and outcome, which are assessed at pre-treatment and during treatment. Second, a sensitivity analysis will be performed under more conservative assumptions (e.g., imputed values of non-completers in the intervention condition will be successively increased, and the analysis described above will be repeated) to investigate the robustness of our conclusions concerning between-group differences.

#### Plans to give access to the full protocol, participant-level data, and statistical code {31c}

De-identified participant-level data as well as analysis code will be made publicly available through a research repository (OSF).

### Oversight and monitoring

#### Composition of the coordinating center and trial steering committee {5d}

The research team reviews recruitment rates weekly and meets on a biweekly basis to ensure the integrity of the protocol and conduct of the study. Any significant amendments to the study protocol will be provided to and approved by the Ethics Committee of Medical School Berlin before implementation.

#### Composition of the data monitoring committee, its role, and reporting structure {21a}

An independent Data Safety and Monitoring Board (DSMB) has been established which will meet once a year. This DSMB is independent of all investigators and the funding agency, and no member of the DSMB has direct involvement in the conduct of the study. The DSMB is composed of three researchers familiar with the area of the study. The DSMB will monitor recruitment, the number of dropouts, and all adverse events including study withdrawals.

#### Adverse event reporting and harms {22}

During the trial, symptom deterioration and suicidality will be closely monitored. Participants in the CETA-I condition who score 1 on the suicide item will be invited to complete the safety module that includes follow-up questions. Participants who indicate immediate plans and means, or previous attempts, will be called and guided through the rest of the safety module. Participants who indicate no immediate plans or means and no previous attempts complete the safety module on their own. Participants who score 2 or above on the suicide item will be called directly and guided through the safety module. (2) Participants will be encouraged to communicate potential adverse effects to their online therapists or the PIs. (3) Online therapists will be required to report adverse events immediately to their supervisor and the PIs. 4) The PI will decide if an adverse event must lead to treatment termination. All such cases will be documented and considered for the analyses of the study results.

At post-assessment, we will collect the negative effects caused by the treatment systematically [57]. Participants will complete the NEQ and are encouraged to further note all adverse events that occurred during treatment in an open-ended question. In addition, other markers indicative of adverse events, like hospitalization, as assessed by FIMPsy, and deterioration rates, will be published.

#### Frequency and plans for auditing trial conduct {23}

The Data Safety and Monitoring Board will audit trial conduct (including recruitment rates and frequency of adverse events) in their yearly meeting.

#### Plans for communicating important protocol amendments to relevant parties (e.g., trial participants, ethical committees) {25}

Any amendments to the study protocol would have to be approved first by the Medical School Berlin Ethics Committee. If approved, these changes would have to be reported in the trial registry. Finally, any amendments would be reported in the trial paper.

##### Dissemination plans {31a}

Trial results will be published in a peer-reviewed journal. The results will also be presented at scientific conferences. A popular science summary of the results will be posted online for laymen and study participants. An anonymized data set as well as statistical code used to analyze the data will be published in a data repository on OSF.

## Discussion

The planned trial aims to develop and evaluate a scalable treatment option for Arabic and Farsi-speaking participants with common mental disorders. We hypothesize that the Internet-based CETA treatment will be more effective than no treatment. The trial will result in important insights about how acceptable this form of treatment is to the target population; how feasible it is for online therapists, supervisors, and patients; and finally how effective it is to decrease the mental health load for patients. We will tailor treatment to the individual patient, an approach that has been successfully realized in previous transdiagnostic online trials [72, 73]. However, the specific tailoring process, including several sources of information (questionnaires, interviews, patients’ problem descriptions), is innovative and has not been tested in an Internet-based setting before. The same applies to the adaptation of the intervention during the treatment process. We know only one Internet-based pilot study that tested just-in-time adaptations for patients with insomnia [74]. Thus, the planned trial not only will provide very relevant data for the undertreated population of refugees but also it will add to the growing field of personalized Internet-based interventions.

### Limitations and conclusion

The planned design is associated with some limitations. First, even if our primary outcome measure is well validated within Arabic and Farsi-speaking populations, we include several questionnaires within our extensive assessment battery that have not yet been translated into Arabic or Farsi. Within a related subproject, careful translations will be performed adhering to WHO standards. However, the results of these questionnaires should still be interpreted with caution as psychometric properties will be largely unknown for the target population. Second, primary and secondary outcomes rely on self-report alone and we do not include a clinician or observer judgment.

Furthermore, acting as a strength and limitation at the same time, we will not apply any exclusion criteria regarding length of stay or asylum status for our patients. This might result in a very heterogeneous group of patients, facing different life situations and different mental health challenges. We will, of course, carefully assess asylum-related factors and evaluate their impact on treatment uptake and outcome. Still, interpretation of (potentially inconsistent) results will be more difficult. We chose these wide inclusion criteria for two reasons. First, it will make it easier to meet our recruitment goal. And second, it will increase external validity. The ultimate goal of the planned project is to help implement efficacious treatments for the undertreated population of refugees and other migrants. By including all participants independent of their time and reason to immigrate to Germany and also by testing the online treatment in other treatment settings in related subprojects, we hope to make a significant contribution to closing the gap between treatment needs and uptake in the vulnerable group of refugees and migrants in Germany.

### Trial status

Participant recruitment starts in November 2023 and is expected to be completed by spring 2025.

### Supplementary Information

**Additional file 1: Appendix 1.** Standard CETA flows.

## Data Availability

After the publication of trial results, a de-identified data set, as well as analysis code, will be made publicly available on the research data repository OSF.

## Abbreviations

ADIS: Anxiety and Related Disorders Interview Schedule
AUDIT: Alcohol Use Disorder Identification Test
CBT: Cognitive behavior therapy
CBT-SQ: Cognitive-Behavioral Skills Questionnaire
CETA: Common Elements Treatment Approach
CETA-I: Common Elements Treatment Approach-Internet-based version
CSQ-I: Client Satisfaction Questionnaire-Internet-based interventions
DIPS: Diagnostisches Interview für Psychische Störungen (Diagnostic Interview for Mental Disorders)
DSM-5: *Diagnostic and Statistical Manual of Mental Disorders-5*
DSMB: Data Safety and Monitoring Board
DUDIT: Drug Use Disorder Identification Test
EQ-5D-5L: European Quality-of-Life 5-Dimensions 5 Level
FIMA: Fragebogen zur Inanspruchnahme von medizinischen und nicht-medizinischen Versorgungsleistungen im Alter (Questionnaire on the Utilization of Medical and Nonmedical Services for the Elderly)
FIMPsy: Fragebogen zur Inanspruchnahme von medizinischen und nicht-medizinischen Versorgungsleistungen bei psychischen Störungen (Questionnaire on the Utilization of Medical and Nonmedical Services for Mental Disorders)
GCTR: German Clinical Trial Registry
GSE: General Self-Efficacy Scale
HSCL-25: Hopkins Symptom Checklist-25
ITT: Intention to treat
NEQ: Negative Effects Questionnaire
OSF: Open Science Framework
OSSS: Oslo Social Support Scale
PCL-5: PTSD Checklist for DSM-5
PHQ-4: Patient Health Questionnaire-4
PMLD: Post-Migration Living Difficulties Scale
PTSD: Post-traumatic stress disorder
RCT: Randomized controlled trial
WAI-I: Working Alliance Inventory-Internet-based interventions
WHO: World Health Organization
