# Supplementary material for: Internet-based transdiagnostic treatment for emotional disorders in Arabic- and Farsi-speaking refugees: study protocol of a randomized controlled trial
Source: Trials. 2024 Jan 2;25:13. doi: 10.1186/s13063-023-07845-5 (PMC10759366; doi:10.1186/s13063-023-07845-5)
Supplement: Supplementary file 1 — Additional file 1: Appendix 1. Standard CETA flows. [file 13063_2023_7845_MOESM1_ESM.docx]

Appendix 1: Standard CETA flows

| Weeks | | Trauma | Depression | Anxiety | Trauma & Depression | Trauma and Substance Use (SU) | Trauma & Depression & SU | Depression & Anxiety | Depression & SU | Anxiety & SU |
| --- | --- | --- | --- | --- | --- | --- | --- | --- | --- | --- |
|  | EP/Intro | EP/Intro | EP/Intro | EP/Intro | EP/Intro | EP/Intro | EP/Intro | EP/Intro | EP/Intro |  |
|  | TDW1 | TDW1 | TDW1 | TDW1 | TDW1 | TDW1 | TDW1 | TDW1 | TDW1 |  |
|  | TDM | GA | LE | TDM | TDM | TDM | LE | GA | LE |  |
|  | TDM | GA | LE | TDM | TDM | TDM | LE | GA | LE |  |
|  | TDM | TDW2 | LE | TDM | TDM | TDM | LE | TDW2 | LE |  |
|  | TDW2 | TDW2/FS | TDW2 | TDW2 | TDW2 | TDW2 | TDW2 | SU1 | TDW2 |  |
|  | TDW2/FS |  | TDW2/FS | GA | SU1 | GA | GA | SU2 | SU1 |  |
|  |  |  |  | GA | SU2 | GA | GA | TDW2/ FS | SU2 |  |
| 9 |  |  |  | TDW2/ FS | TDW2/ FS | TDW2 | TDW2/ FS |  | TDW2/ FS |  |
| 10 |  |  |  |  |  | SU1 |  |  |  |  |
| 11 |  |  |  |  |  | SU2 |  |  |  |  |
| 12 |  |  |  |  |  | TDW2/ FS |  |  |  |  |

E/Intro = Psychoeducation & Engagement, TDW= Thinking in a Different Way (Cognitive Restructuring), TDM = Talking about Difficult Memories (=Trauma Exposure), FS = finishing Steps (relapse prevention), GA = Getting Active (behavioural activation), LE=Life exposure (imaginal and in-vivo exposure), SU=Substance Use. Standard flows will be adapted to the individual patient.
During treatment, flows can be adapted by a) adding substance use modules, safety planning (SP), and problem solving (PS) when issues arise and b) increase dosage by a maximum of 0.5 sessions for TDWI & EP/intro and by a maximum of 2 sessions for GA, TDW II, TDM, LE, SU, and PS.
